# Settling Ulysses: An Adapted Research Agenda for Refugee Mental Health

**DOI:** 10.15171/ijhpm.2017.131

**Published:** 2017-11-08

**Authors:** Yudit Namer, Oliver Razum

**Affiliations:** Department of Epidemiology and International Public Health, School of Public Health, Bielefeld University, Bielefeld, Germany.

**Keywords:** Ulysses Syndrome, Refugees, Asylum Seekers, Mental Health, Belonging

## Abstract

Refugees and asylum seekers arriving in Europe during the 2015/2016 wave of migration have been exposed to war conditions in their country of origin, survived a dangerous journey, and often struggled with negative reception in transit and host countries. The mental health consequence of such forced migration experiences is named the Ulysses syndrome. Policies regarding the right to residency can play an important role in reducing mental health symptoms. We propose that facilitating a sense of belonging should be seen as one important preventive mental healthcare intervention. A refugee mental health agenda needs to take into account the interplay between refugees’ and asylum seekers’ mental health, feeling of belonging, and access to healthcare. We urge for policies to restore individuals’ dignity, and recognize the right for homecoming to parallel the mythology of Ulysses.

The mental health-related aftermath of forced migrants’ tumultuous voyage to a foreign land has been named the Ulysses (or Odysseus) syndrome by Joseba Achotegui, a cultural psychiatrist working with migrants in Spain.^1^ The syndrome comprises an atypical depressive presentation with anxiety-related somatoform and dissociative symptomatology, and migratory grief.^2^ Symptoms may include migraines, generalized worry, gastric and other physical pains, irritability, and insomnia.^1^ The symptoms are exacerbated by continuing separation from one’s country of origin, by cultural and social segregation, and by the pressure to achieve certain immigration goals either personally or officially set.^2^ Introduced to describe the difficulties faced mostly by Latin American migrants in Spain, interest in the Ulysses syndrome has resurfaced following the recent wave of refugee migration to Europe.^3^

There is of course substantial heterogeneity among tales of migration. Nonetheless, most individuals arriving in Europe during the refugee migration in 2015/2016 have been exposed to oppression or war conditions, just like Ulysses, the forced migrant protagonist of the Odyssey who wandered for 10 years following the Trojan War.^4^ Transit to Europe has also been traumatic, with policy and politics adding insult to injury. Again, like Ulysses, refugees and asylum seekers have encountered ambivalent and at times hostile reception in Europe. Their arrival has been complicated by the physical or symbolic loss of significant others; housing-, employment- and documentation-related difficulties; and in some cases, detention and threat of deportation.^5^ Ulysses had the chance to ameliorate his condition quickly and was able to make a home again. Not so the refugees: they experienced little if any feeling of belonging.

Seeking to belong as a primary human motivation is an established phenomenon in psychological literature. Baumeister and Leary argue that belonging can be considered a fundamental human motivation for the following reasons: it functions under many conditions and it involves emotional and cognitive processes. Its obstruction leads to negative consequences such as psychological and physical ailments; it is universal and non-derivative of other motivations; and it has significant implications for psychological operations. They further argue that communities utilize belonging to reward and reprimand members: exclusion in the form of imprisonment, exile or encampment is used by many cultures to punish.^6^ Indeed, sense of belonging has been characterised to incorporate the recognition of being valued by one’s social environment, and the recognition of congruency with others in the environment.^7^ Relatedly, sense of belonging was demonstrated to be a crucial component of mental health.^8^

Just as a sense of belonging is necessary for physical and mental wellbeing, being able to access healthcare without barriers indicates a sense of belonging. We believe that one indicator of congruency, that is a sign of inclusion, is being able to receive treatment for health problems with the same degree of access as all members of the community. There is without doubt a need for care: refugees and asylum seekers experience higher rates of posttraumatic stress disorder, anxiety and depression.^9^ Such findings justify the growing efforts to develop valid screening methods to identify symptomatology, as well as safe, empirically sound, culturally-appropriate and effective mental health interventions. For example, a recent systematic review^10^ of psychosocial interventions developed for or used with refugees and asylum seekers indicates narrative exposure therapy^11^ to have evidence-based suitability for refugees with posttraumatic symptomatology. Yet mental healthcare is generally not prioritized, neither by governments nor by donors.^12^ In parallel, what is missing from public health literature are data regarding the extent to which refugees and asylum seekers have access to these interventions.

Level of access to healthcare across Europe varies considerably. In Germany, for example, refugees and asylum seekers are entitled solely to vaccinations, emergency and maternal healthcare in the first 15 months of arrival; other care, such mental healthcare is subject to formal request.^13^ Yet, compared to residents, asylum seekers in Germany are less likely to see physicians but more likely to consult psychotherapists when access becomes possible, presumably reflecting higher, and unmet needs.^14^ As long as empirically sound interventions are not included in healthcare entitlements, are too difficult to access, or are not developed for the target culture in mind,^12^ research efforts will not translate into a feeling of belonging for refugees and asylum seekers. In other words, when cultural barriers create a gap between the need for care and the available effective treatment, and when mental healthcare is available to residents/citizens but not to refugees and asylum seekers, congruency with others in the environment that is necessary for the sense of belonging to emerge will not materialize.

Access to healthcare is not the only policy- and politics-related problem that compromises refugees’ and asylum seekers’ feeling of belonging. Post-displacement conditions, repatriation status and the stage of conflict in place of origin (where one fears returning) moderate refugees’ mental health.^15^ Immigration detention is found to significantly contribute to posttraumatic stress and depressive disorders, and this effect appears to persist for years after release.^16^ Asylum interviews can also impact trauma-related symptoms; a decrease in hyperarousal and posttraumatic avoidance and an increase in posttraumatic intrusions in applicants were found weeks after the interviews.^17^ In one study, refugee community advocates described asylum interviews as akin to torture and interrogations subjected in the country of origin, and thus causing “emotional paralysis.” One advocate indicated in addressing the asylum waiting process, “People have lost their human dignity.”^18^ In terms of Baumeister and Leary’s conceptualization,^6^ the message of detentions and interrogative asylum interviews, with the threat of deportation always present in the room, is that of punishment by exclusion, a clear statement of not belonging.

As these findings suggest, shifting the restrictions on entitlements and removing access barriers as advocated by public health research,^13^ although steps in the right direction, would not by themselves improve mental health. An embodied illustration comes from Sweden, where asylum-seeking children have analogous access to healthcare as resident children. Beginning in 2003, a significant number of children facing threat of deportation received a diagnosis of *Uppgivenhetssyndrom*, characterized by a state of severe apathy, stupor, non-communication and loss of bodily functions.^19^ It was later found that these children had biomarkers of severe long-term stress.^20^ A multifactor explanatory model asserted the asylum process, long waiting times and experience of rejection by the host country to be among the triggering factors. Although not immediate, among the healing factors were gaining residence rights and the renewed sense of hope it brought.^21^ Evidence suggests that acquisition of residency – a clear signal towards belonging – indeed results in betterment of mental health: it significantly reduced refugees’ posttraumatic stress, anxiety and depression scores in the Netherlands through an improvement in living conditions.^22^

From a public health and epidemiological perspective, it is difficult to establish the contribution of policies and politics to the trauma symptomatology observed in refugees and asylum seekers because of the different levels of analysis (when the exposure is a national policy and the outcome individual health, only between-country comparisons can be informative; these, however, are likely to suffer from confounding by differences between respective political systems as well as refugee populations). It may not even be possible to speak of *post*-traumatic symptomatology when deterrence prevails in national policies. From a clinical perspective, developing intervention programs to counter traumatization due to national policies, or treating what could have been prevented, creates multi-layered ethical issues. Adopting a community-based public mental health approach may generate positive change not only for refugee populations, but also for the host societies. It would focus not on illnesses and symptoms but on ecological and transgenerational resiliencies,^23^ and all institutions would take part in upstream prevention. Such models are usually developed for post-conflict zones and humanitarian emergencies. However, in the case of refugee migration to Europe, community-based approaches may not only strengthen the resilience of refugees, but may contribute towards building heterogeneous yet cohesive societies. When it comes to policy research efforts, best practice would involve interdisciplinary, multi-actor efforts evaluating the interplay between refugees and asylum seekers’ mental health, feeling of belonging, and access to healthcare. The ethical and scientifically responsible path is to involve all participants of healthcare systems, including refugees and asylum seekers themselves, to propose policy that promotes belonging as preventive healthcare. The UK-based mental health charity Mind specifically recommends the incorporation of refugee community organisations and mental health advocacy training.^18^

For refugees and asylum seekers, going “home,” returning to their personal Ithaca, may not be possible. This loss is mourned over every day. Yet, what eventually instilled in Ulysses the sense of homecoming was not coming home *per se*, as he was not recognized on his initial arrival, but the eventual recognition and the reassertion of his dignity. In a global era characterized by travel bans, closed borders and “refugee deals,” the tale of Ulysses should inspire policymakers and politicians alike to instil the feeling of belonging in refugees and asylum seekers by granting the right to health. This implies granting entitlements to healthcare equal to those of the majority populations of host countries, and lifting access barriers not only to healthcare, but also blocking access to employment, residency and public life. In other words: restore individuals’ dignity, and recognize the right for homecoming, as soon as possible. Otherwise, healthcare systems will once more be left with the task of treating the health consequences of political decisions.

## Ethical issues

Not applicable.

## Competing interests

Authors declare that they have no competing interests.

## Authors’ contributions

Both authors conceptualized the perspective. YN drafted the first version of the manuscript. OR critically revised the manuscript for important intellectual content. Both authors read and approved the final manuscript.
